# A stakeholder co-design approach for developing a community pharmacy service to enhance screening and management of atrial fibrillation

**DOI:** 10.1186/s12913-018-2947-7

**Published:** 2018-02-27

**Authors:** Daniel Sabater-Hernández, Jacqueline Tudball, Caleb Ferguson, Lucía Franco-Trigo, Lutfun N. Hossain, Shalom I. Benrimoj

**Affiliations:** 1 Graduate School of Health, University of Technology, Level 4, Building 7, 67 Thomas St, PO Box 123, Ultimo, Sydney Australia; 2 Academic Centre in Pharmaceutical Care, University of Granada, Granada, Spain

**Keywords:** Atrial fibrillation [MeSH], Self-monitoring, Health services [MeSH], Community pharmacy services [MeSH], Co-design, Health planning [MeSH]

## Abstract

**Background:**

Community pharmacies provide a suitable setting to promote self-screening programs aimed at enhancing the early detection of atrial fibrillation (AF). Developing and implementing novel community pharmacy services (CPSs) is a complex and acknowledged challenge, which requires comprehensive planning and the participation of relevant stakeholders. Co-design processes are participatory research approaches that can enhance the development, evaluation and implementation of health services. The aim of this study was to co-design a pharmacist-led CPS aimed at enhancing self-monitoring/screening of AF.

**Methods:**

A 3-step co-design process was conducted using qualitative methods: (1) interviews and focus group with potential service users (*n* = 8) to identify key needs and concerns; (2) focus group with a mixed group of stakeholders (*n* = 8) to generate a preliminary model of the service; and (3) focus group with community pharmacy owners and managers (*n* = 4) to explore the feasibility and appropriateness of the model. Data were analysed qualitatively to identify themes and intersections between themes. The JeMa2 model to conceptualize pharmacy-based health programs was used to build a theoretical model of the service.

**Results:**

Stakeholders delineated: a clear target population (i.e., individuals ≥65 years old, with hypertension, with or without previous AF or stroke); the components of the service (i.e., patient education; self-monitoring at home; results evaluation, referral and follow-up); and a set of circumstances that may influence the implementation of the service (e.g., quality of the service, competency of the pharmacist, inter-professional relationships, etc.). A number of strategies were recommended to enable implementation (e.g.,. endorsement by leading cardiovascular organizations, appropriate communication methods and channels between the pharmacy and the general medical practice settings, etc.).

**Conclusion:**

A novel and preliminary model of a CPS aimed at enhancing the management of AF was generated from this participatory process. This model can be used to inform decision making processes aimed at adopting and piloting of the service. It is expected the co-designed service has been adapted to suit existing needs of patients and current care practices, which, in turn, may increase the feasibility and acceptance of the service when it is implemented into a real setting.

**Electronic supplementary material:**

The online version of this article (10.1186/s12913-018-2947-7) contains supplementary material, which is available to authorized users.

## Background

Atrial fibrillation (AF) is one the most common heart rhythm disorder. Globally, it is estimated that 30 million individuals are diagnosed with AF [1]. Cardiovascular morbidity and mortality related to AF remains high and is primarily associated with cardiovascular death, heart failure, stroke and hospitalisation [1]. Individuals with AF have a three to five-fold risk of developing stroke [2] with AF estimated to be a contributing factor of 25–30% of all ischaemic strokes [3]. AF-related strokes are often more catastrophic and disabling than strokes of other origin [4]. However, in the majority of cases these strokes are preventable, since in more than 25% of cases, the stroke was the first indicator of a previously unidentified AF. Prevention is two-fold, reliant on timely AF diagnosis and optimisation of thromboprophylaxis [5].

Internationally, the proportion of undiagnosed AF is estimated to be between 11% and 30% [3] ,which evidence an obvious need for systematic community-based screening programs. In 2016, the Atrial Fibrillation Network, the European Heart Rhythm Association, and others, have recommended the establishment of widespread screening programs for AF in individuals aged over 65, and those at high risk, including stroke survivors [1]. To date, much attention has been directed towards promoting opportunistic AF screening and developing systematic screening programs. However, limited attention has been paid to the area of patient self-monitoring and -screening of AF, which is a suitable, yet underused practice [6]. Given the availability of blood pressure (BP) monitoring devices that integrate reliable algorithms to detect AF one could postulate that prevention strategies may include the concept of patient self-monitoring and screening [7–9]. Home BP monitoring is a widely acknowledged practice adopted by many hypertensive patients that enables multiple measurements of BP over extended periods of time [10, 11]. Compared to an opportunistic single-screen at one point in time, self-monitoring for BP/AF at home potentially increases the chance of detecting AF. Therefore, adopting automated devices for BP monitoring that integrate reliable algorithms to detect AF is a promising practice that may enhance the diagnosis and management of AF and thus reduce AF-related cardiovascular morbidity and mortality in the long term [9].

Community pharmacies offer a broad and accessible network of health facilities at the primary care level that provide access to a large number of individuals at risk who may be screened for a range of conditions [12]. Additionally, there is evidence that community pharmacy services (CPSs) can positively influence patients’ behaviours, such as self-monitoring of BP [13, 14], and, eventually, cardiovascular outcomes [15]. To date, previous studies exploring AF-screening services in the community pharmacy have used a single, in-pharmacy opportunistic screening by a pharmacist as the principal detection method [16, 17], yet no pharmacist-led CPSs that promote self-monitoring of AF as the main screening method have been found in the literature. Developing, implementing and evaluating novel CPSs are complex challenges [18], which require comprehensive planning and participation of relevant stakeholders [19, 20].

Co-design processes are participatory research approaches that engage service users, healthcare professionals and any other key stakeholders with a vested interest in a particular problem in developing (and further implementing and evaluating) health services [21, 22]. Having stakeholders working collaboratively brings several benefits to the service planning process that can eventually facilitate their adoption into practice [23, 24]. Generally, it fosters co-learning, nurtures networking, develops stakeholders’ positive feelings of ownership on the planned service, and brings innovative ideas, logistical and financial support. In their early stages, co-design processes require stakeholders to reflect on their own healthcare experiences, working together to identify relevant needs and priorities for improvement and to devise strategies to address the identified issues [22]. This facilitates not only an appropriate understanding of patients’ needs and existing gaps in healthcare, but also of those circumstances that can enable or hinder the delivery and implementation of the service. As a result, co-designed health services are adjusted to the specific context or environment in which they are to be integrated, which, in turn, increases their acceptability and feasibility [25]. Co-design processes have been used in a broad range of healthcare settings adopting diverse research approaches, generally qualitative [26–29]. However, co-design has been infrequently employed in the pharmacy setting, despite the potential convenience as per existing service development and implementation challenges. Hence, the aim of this study was to use a stakeholder co-design approach to develop a pharmacist-led CPS aimed at enhancing self-management of AF (by promoting self-monitoring and screening).

## Methods

A 3-step co-design process using qualitative methods (i.e., focus groups and individual interviews) was conducted to develop the aimed service. Overall, the process sequentially engaged: (1) potential service users to understand their needs and concerns and inform the next steps, (2) a mixed group of service stakeholders to work collaboratively and generate a preliminary model of the service and (3) a group of community pharmacy owners and managers to initially explore the feasibility and appropriateness of the co-designed model.

1. *Step 1: Exploring the views of potential service users.* A focus group and semi-structured interviews were conducted with individuals over 65 years with hypertension and/or AF. The focus group lasted 2.5 h and involved 4 individuals. Interviews were conducted face-to-face (*n* = 2) or by phone (*n* = 2). The focus group and interview guide is provided in Additional file 1. All participants were fluent in English. Individuals with a cognitive impairment, an intellectual disability or a mental illness were excluded. The aim of both the focus group and interviews was to explore: (1) the knowledge/awareness, concerns and needs of participants regarding AF, (2) their experiences with AF, hypertension (including management and monitoring) and health services; and (3) their opinions about community pharmacists and a hypothetical CPS aimed at self-screening/monitoring of AF. This latter part included specific discussions about the device Microlife BP A200 AFIB (Microlife Corp., Taipei, Taiwan), which is a clinically validated BP measurement device that integrates an algorithm for the evaluation of pulse irregularity and the detection of AF during automated BP measurement [7–9]. The educational material developed by the company that has the rights to distribute the device in Australia and funded this research was assessed for appropriateness. Participants were recruited through existing networks and a not-for-profit association that organises social activities for the elderly.
2. *Step 2: Delineating a preliminary model of the service.* A 4.5-h focus group with a mixed group of stakeholders with a vested interest in the aimed CPS was conducted. Stakeholders included 8 participants who could directly or indirectly participate or have an influence in the service, including: 1 potential service user from the previous group; 2 community pharmacy owners/managers/service providers; 1 general practitioner (GP); 1 cardiologist; 1 heart failure nurse practitioner; 1 research nurse with expertise in AF; and 1 representative from the Australian Stroke Foundation. The aim of this focus group was to delineate a preliminary model of the CPS. The findings of the previous step were used to inform the group discussions, which sequentially addressed: (1) the target population of the service (i.e., Which patients would most benefit from the service?); (2) the service components, including assessments by the pharmacists, interventions targeting patients and healthcare providers, patient referral and follow-up processes (i.e., How does a high-quality pharmacy service for the targeted population look like?); and (3) what are the circumstances that can enable or hinder the delivery or implementation of the service in the existing context (i.e., what should be taken into account to integrate the service into practice?). Between the second and third part of the discussion, participants were given the educational material for assessment. The focus group guide is provided in Additional file 2. Stakeholders were purposely selected and contacted by the research team to bring a broad range of individuals and organizations that allowed for a large expertise and experience and so for designing a comprehensive service that suits current needs of patients and the healthcare system.
3. *Step 3: Initial assessment of the feasibility and appropriateness of the co-designed model.* A 2-h focus group with 4 community pharmacy owners and managers was conducted. The aim of this focus group was to further explore the feasibility and appropriateness of the CPS and assess the professional and economic implications of such a service from a business perspective. Participants were provided with an overview of the previously delineated model of the service and asked for their views about such a model. The same sequence, topics and questions delineated for the previous focus group was used (Additional file 2). Pharmacists were purposely selected and directly contacted by the research team due to their previous experience in CPS delivery.

All focus groups and interviews were facilitated by a researcher (JT) with experience in qualitative research. Focus groups took place within the university setting. Individual interviews were held in a private meeting room on campus, or were conducted by phone, according to the wishes of participants. Interviews and focus groups were audio-taped and transcribed verbatim. Ethical approval for this study was obtained by the Human Research Ethics Committee of the University of Technology, Sydney (reference number: ETH16–0276). All participants were appropriately informed about the study and written consent was obtained.

### Data analysis

Data were managed in QSR NVivo (10) and analysed using Bazeley’s framework of ‘describe-compare-relate’ to identify themes and intersections between the themes [30]. Data were analysed descriptively (i.e., categories were formed directly from participant’s responses) by one researcher (JT) and reported to the co-investigators (DSH and CF) for their comment. Subsequent analyses “challenged, extended and supported” the descriptive nodes and focused on concepts emerging from observation of all data at the more abstract level and on developing themes from these concepts. This later stage of analysis involved cycles of analysis whereby coding was reflected upon critically as new themes were developed and revised if necessary. Developing themes were compared to each other and relevant associations and differences were recorded. Themes were also compared between different focus groups and in relation to occupation and background of participants. Data from the two focus groups were analysed together in an additional analytical process. Results of this process were organised using the same structure as in the focus group: (1) potential target population, (2) service components, and (3) circumstances that may affect service implementation. To assist in the identification of these latter circumstances the list determinants of practice described by Flottorp et al... (i.e., Tailored Implementation in Chronic Disease checklist) was used [31].

Eventually, to organise the analysed data and build a theoretical model of the service, the JeMa2 model to conceptualize pharmacy-based health programs, as described by Sabater-Hernández et al [20], vvpharmacist interventions/pharmacy services can improve individuals’ health and quality of life by immediately affecting the determinants of both behavioural and environmental causes of health. Moreover, the model hypothesizes the relationships between pharmacy services and the context in which such services are implemented. It envisages the circumstances that can influence the implementation of the service into routine practice and the set of strategies and interventions aimed at supporting such implementation. The end result is the pharmacy-based health program comprised of the pharmacy service and the strategies and interventions that support its implementation, sustainability and overall impact [20].

## Results

### Exploring the views of potential service users

The range of age of the 8 individuals (7 women) who participated in the focus group (*n* = 4) and the interviews (*n* = 4) varied between 70 and 88 years old. Three participants had a diagnosis of AF (an additional one was unsure); including 2 with an implanted pacemakers, and 5 had hypertension (2 participants with both AF and hypertension). One had neither condition, but had a family history of hypertension. Four main categories relevant to the research questions were identified from the potential service user dataset:

- *Awareness and knowledge of AF.* Low awareness and knowledge of AF was apparent among participants who had AF and was non-existent among those who did not. Indeed, one participant who had a pacemaker was uncertain why she had it, but was sure that her doctors had never used the term ‘atrial fibrillation’ in discussions with her. The advertising materials that were designed to increase awareness and knowledge of AF were considered to be counter-productive for that purpose, because of the negative cast of the language used and the lack of explanation for the medical terminology of ‘atrial fibrillation’ (Q1; Additional file 3).
- *Emotional distress.* Emotional distress was found to operate in two ways with regards to AF. Firstly, participants with AF reported that AF symptoms are very similar to the physical experience of emotional distress and so it is difficult to distinguish between ‘being stressed’ and ‘going into AF’. Differentiating the cause of certain physical symptoms of AF, such as a ‘racing heart’, from the physical experience of ‘stressful life circumstances’ – which could also result in an increased heart rate – was particularly challenging (Q2; Additional file 3). This had consequences for how they responded to and managed these symptoms, as emotional stress could be managed by relaxation techniques, yet AF may sometimes require medication or medical intervention, dependant on type of severity. Secondly, emotional distress as a result of frightening or unpleasant symptoms of AF could be a motivating factor in learning more about the condition and preparing for a future AF event. The degree of emotional distress AF symptoms caused participants appeared to influence their awareness of the condition, particularly regarding future AF attacks (Q3; Additional file 3) Those who had experienced particularly frightening symptoms when in AF were somewhat more aware about their condition and prepared for its consequences, such as having a plan for an AF episode (Q4; Additional file 3).
- *Perception of self-monitoring.* One participant with AF considered a benefit of having a monitoring device was it helped to differentiate whether emotional distress was due to AF or other causes (Q5; Additional file 3). Most participants with AF had experienced few symptoms (ever or recently) and believed that their condition was currently well-managed. Thus, AF did not have a significant impact on their lives and monitoring the condition was of low priority (Q6; Additional file 3). Those who had hypertension felt similarly given the good control they had achieved with medication only, although they could see a need if they were worried or when their BP was not well-controlled (Q7; Additional file 3). Two participants who did not have AF or hypertension expressed concerns about being over-serviced if they engaged with self-monitoring (Q8; Additional file 3). Participants were unconcerned by the possibility of AF being detected at home, where a health professional is not immediately present to explain the findings. They were experienced with diagnostic procedures and held sufficient trust in their doctors and pharmacists to await their input before worrying or acting on the information provided by the device (Q9; Additional file 3).
- *Role of community pharmacists and pharmacy services in in identifying and managing AF.* There was widespread support for pharmacists to assist with the monitoring of AF. However, participants were explicit and unanimous in their view that pharmacist engagement in the management of AF must be in partnership with GPs, who should also recommend and support self-monitoring (Q10; Additional file 3). It was also important that participants’ privacy was maintained as a matter of principle and that information sharing between pharmacies and GPs must be conducted only with their explicit permission (Q11; Additional file 3). The fee for the service needed to be what participants considered to be ‘reasonable’ and should vary according to the individual level of government-funded benefits (i.e., aged pension) and the payment fees/conditions of services they currently access. Participants who had no prior experience of monitoring their BP or were particularly concerned about hypertension were willing to pay a nominal fee of “*up to $25*” AUD. However, some participants highlighted that BP screening services were ‘already free’, so they considered it unreasonable to charge an excessive fee to explore for just one additional condition in addition to BP (Q12; Additional file 3).

### Building up the model of the service

#### Potential target population

There was a high level of agreement among stakeholders as to whom the potential users of the pharmacy service might be. It was quickly established that those individuals who were 65 years or older, with hypertension, with or without existing AF, and with or without a previous history of stroke would be the priority target group (Q13; Additional file 3). It was noted that the service could be rolled out to Aboriginal & Torres Strait Islander people as the second tier of implementation.

#### Service components

Stakeholders defined three main components of the service:

- *Patient education.* The low awareness and knowledge of AF among high risk individuals concerned many stakeholders, who considered education to be a fundamental component of the service. However, it was highlighted that close attention to the language of education materials was required so that it was understandable and meaningful to the majority of patients, taking account of varying levels of health literacy. Responses from participants in all three focus groups and interviews noted the need to avoid ‘medicalised’ language, encourage the use of plain language, and explain key terms in the current educational materials developed by the device manufacturer (Q14; Additional file 3). Stakeholders highlighted the need to use different methods, techniques (e.g., pharmacists providing in-situ demonstrations on how to use the device; patient self-learning by taking information home) and materials (e.g., booklets, information sheets, websites, videos, podcast) in acknowledgement of different learning styles (Q15; Additional file 3). Furthermore, educational material for patients should be endorsed by leading cardiovascular organizations (including the heart and stroke foundations and national cardiac societies and colleges).
- *Self-monitoring of AF/BP at home.* Participants in the mixed stakeholder focus group highlighted the importance of implementing reliable programs that follow acknowledged recommendations for self-monitoring and employ accurate devices. They were wary of the reliability of current self-monitoring practices given the poor accuracy and performance of many BP monitoring devices available to patients on the market. Self-monitoring was suggested to be conducted for 2–4 weeks (Q16; Additional file 3). Patients must understand the device is not a diagnostic tool and an electrocardiogram (ECG) must be conducted when a positive AF screen is detected (Q17; Additional file 3). Pharmacists in the third focus group agreed on the inclusion of the self-monitoring component and were adamant that an isolated screening service in the pharmacy would not be successful. They mentioned that few patients felt a personal need/interest for AF screening and so it was difficult to charge a fee. This makes isolated AF/BP screening in the pharmacy an uneconomic exercise (Q18; Additional file 3). Self-monitoring of AF/BP was deemed different and more valuable than solely in-pharmacy screening and that might facilitate the remuneration of the service (Q19; Additional file 3).
- *Evaluating results, referral and follow-up.* In-pharmacy consultations were proposed by the mixed group of stakeholders to ensure self-monitoring occurred, and to process and interpret the results. Pharmacy owners/managers acknowledged the value of downloading, processing and discussing the results with patients. They also saw the value of preparing robust reports derived from reliable, trustworthy technology and using technical language to communicate disease progress to GPs and other healthcare providers. However, they were initially doubtful of the potential for success of these follow-up consultations since, normally, once a device is sold, customers tend not return to the pharmacy (Q20; Additional file 3). When AF or high BP is detected a referral should occur with the possibility of the pharmacist assisting by making the appointment on behalf of the patient. The need to follow-up patients after being referred to the GP was also suggested, so as to check whether patients attend the appointment or any decision was made as a result of the service (Q21 and Q22; Additional file 3).

#### Circumstances that may enable or hinder the delivery or implementation of the service (i.e., determinants of pharmacy practice)

**Fig. 1:**
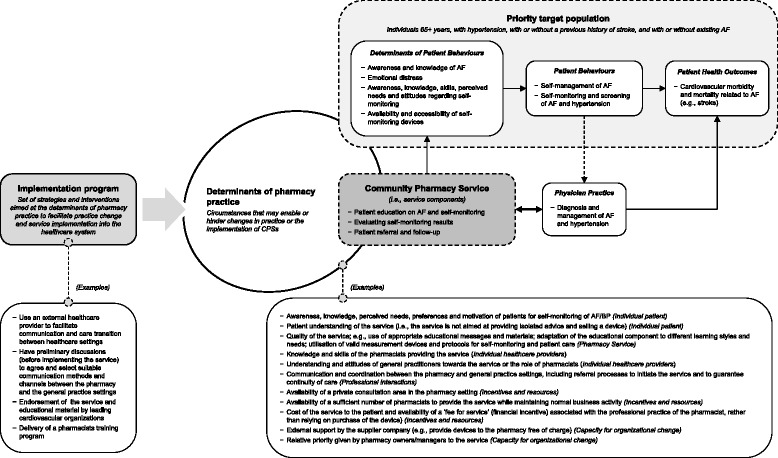
Theoretical model of the co-designed community pharmacy service

Apart from some key issues already identified above (e.g., quality of the service, cost of the service to the patient, etc.), five main themes arose from the analysis of the stakeholder data (see all the identified determinant of pharmacy practice in Fig. 1):

- *Service provider and qualification.* It was agreed by all stakeholders that the service must be provided by a pharmacist with appropriate qualification and should not be delegated to a lesser-qualified staff member. This would lend the service the necessary professionalism and legitimacy to allow consumers to have confidence in the quality of service (Q23; Additional file 3).
- *Privacy in the pharmacy setting.* All stakeholders agreed on the need for a private consultation area to provide the service, which also allows for a suitable environment to perform an accurate BP reading (Q24; Additional file 3). This need also aligned with patients’ views on the importance of privacy and confidentiality.
- *Pharmacy staffing.* Pharmacies needed to be staffed by a sufficient number of pharmacists at any given time so that the general business of the pharmacy could continue whilst the service is provided (Q25; Additional file 3). This may be difficult for small, independently-owned pharmacies in which a private consultation room may not exist. However, there was concurrence across both stakeholder focus groups that the aforementioned type of pharmacies would be better placed to provide the service, as they were considered to have a commitment to health service provision over and above profit (Q26; Additional file 3).
- *Service remuneration.* Stakeholders agreed the fee for service should not be associated with the sale of the device only, since not all services would progress to sales and the profit margin is relatively low. It was suggested that patients could pay a fee for both renting the device and for the service of professional advice. Renting the device was seen as low-cost alternative to immediate purchase that allows patients to testing the device and assessing its usefulness. Ultimately, this rental models might lead to a greater number of sales (Q19; Additional file 3). Pharmacists in the pharmacy focus group also suggested that the manufacturer should provide devices free of charge to pharmacies to establish the service.
- *Inter-professional relationships.* Effective communication between pharmacies and general practices were emphasised as being especially important by all stakeholders. Regular communication with GPs was emphasised particularly by doctors as a requirement to maximise the success of the service. They recognised that well-established communication channels were important, but there were individual differences in preferred modes of communication. Thus, communication methods would need to be formalised and agreed upon prior to the roll-out of the service (Q27 and Q28; Additional file 3). Pharmacists anticipated low levels of success in communicating or collaborating with GPs due to misunderstandings regarding professional boundaries and the misinterpretation of the actions of pharmacists (Q29; Additional file 3). They also suggested that service users do not find it necessary for pharmacists to share information with GPs and many customers prefer to manage the information exchange themselves (Q30; Additional file 3). However, potential service users alluded to times when it would be advantageous for their GPs and pharmacists to discuss their situation without acting as the ‘go-between’ (Q31; Additional file 3). The mixed group of stakeholders suggested establishing regular visits to the pharmacy by a nurse practitioner to deliver the service or facilitate coordination/communication between healthcare settings (Q32; Additional file 3). However, pharmacists in the pharmacist focus group did not support this suggestion.

Based on the JeMa2 model, all the information obtained through the co-design process was organised to create a pictorial representation of the CPS. As illustrated in Fig. 1, the depicted model delineates the pharmacy service along with the causal chain that explains how it is expected to work and generate its impact on the defined target population. These are the relationships between the specific components of the service; the targeted determinants; the expected changes in patients’ and physician practices; and the final health outcomes of the service. Finally, the model recaps the range of circumstances that were posed to influence the implementation of the service, along with few strategies that were advised to address some of those issues.

## Discussion

This study summarises a 3-step co-design experience that resulted in the development of a preliminary model of a CPS aimed at encouraging self-monitoring/screening of AF. This is considered a relevant first step to suitably integrating community pharmacists with the management of AF and hence taking advantage of the accessibility of such a setting to reach a larger number of individuals in need. Importantly, the co-design process integrated the concerns, expectations and values of patients, along with the cumulated experience, knowledge and skills of key healthcare professionals, who may implement, deliver or interact with the service. As a result, it is expected the co-designed service has been adapted to suit existing needs of patients and current care practices, which, in turn, may increase the feasibility and acceptance of the service when it is implemented into a real setting.

The co-design process was predominantly driven by the self-monitoring component, which provided the original fundamentals for developing the new service and was established as the central service component from the outset. Overall, participant stakeholders agreed on the potential role of pharmacists in encouraging self-monitoring/screening practices and pursued to clearly understand how they would be adequately integrated within the pharmacy setting to enhance current AF care. Moreover, as in a previous study [32], patients stated they would feel comfortable to self-monitor their condition and then report the results to a healthcare professional. A good example about how the self-monitoring component drove the discussions in the co-design process can be found when defining the target population. Participants stakeholders considered the target population should have an anticipated interest, motivation or need for self-monitoring and so to receive the service. Based on that argument, stakeholders prioritised individuals with existing hypertension as the target population. Also, due to the increased risk of AF of hypertensive individuals [33], it was argued the service’s AF detection rates and thus its efficiency may be increased. Overall, the combination of both a better approach for AF screening (based on repeated measures) and a more specific target population may enhance the acceptance and feasibility of a service and overcome certain implementation challenges compared to previous, isolated AF screening programs in the community pharmacy targeting broader populations [16, 17, 34, 35].

Patient education also emerged as a core component of the co-designed service. This is consistent with existing literature on educational needs in AF, which acknowledges patient education lays the foundation to change patient’s attitudes, behaviours and so achieve better outcomes and must address the two key challenges reported in this study (i.e., enhance awareness/knowledge about AF and manage the physiological component associated with AF) [36]. Besides, given the self-monitoring component, patient education also becomes relevant to ensure the quality of self-monitoring [37], which is an acknowledged international concern [10, 11, 38]. Education in this regard must address the suitable recommendations for self-monitoring [10, 11]. Importantly, additional educational efforts must be directed to increasing awareness and uptake of AF screening among patients for whom self-monitoring is warranted, yet they are not fully cognisant of the benefits of the practice to their individual circumstances.

In this co-design process, the JeMa2 model was used to organise the gathered information and construct the theoretical model of the CPS. The model allows for a comprehensive depiction of the CPS beyond the components of the service [20]. Firstly, the model encompasses the set of behaviours (i.e., patients’ and physicians’ practices) and determinants of patients’ behaviours that are immediately targeted by the CPS. These intermediate variables show the ‘casual links’ that explain how the CPS is expected to improve health and inform the changes that must be assessed for the impact evaluation of the CPS [39]. Secondly, the model addresses the context in which the CPS will be implemented, including the social, physical, economic, and policy environments. Assessing and identifying the circumstances that can positively or negatively influence implementation from the outset of the health service planning process is crucial for the early adaptation of services (i.e., adoption of best methods, procedures or materials) to best fit existing healthcare practices and also for informing the development of strategies and interventions to support the implementation of the service into practice [25, 40].

It should be noted that despite the comprehensive approach adopted by the JeMa2 model, the model generated from this process is limited by the number of stakeholders (19 in total) who participated in an, equally restricted, number of co-design steps. Therefore, the resulted model must be seen as a preliminary approach that requires further development and decision-making before being piloted in a real setting. For example, at the service level, discussions, agreements or research are required to define a clear self-monitoring schedule for detecting AF, or patient referral or follow-up criteria. To date, guidance for recommendations to optimal method, location and frequency of AF screening are not well described within international AF guidelines. Therefore, current recommendations for BP can be used as a starting point [10, 41]. Also at the service level, a need for conducting additional research to inform the adoption of educational methods, techniques and materials that can suit stakeholder’s needs, preferences, etc. is acknowledged. At the contextual level, the list of circumstances that may influence service implementation is not comprehensive and further groundwork and research is needed to ensure all key circumstances have been identified. Previous theory and research on determinants of healthcare practice [31, 42] and, specifically, on barriers and facilitators to CPS implementation [43, 44] may assist in this regard. Similarly, additional work will be necessary to identify, select and precisely define the implementation strategies and interventions once the most relevant determinants of practice have been determined [45, 46].

## Conclusions

This co-design process generated a preliminary, yet novel model of a community pharmacy-based health program that envisages the integration of community pharmacists in the management of AF; particularly, by promoting self-monitoring/screening of AF. As a result of integrating the views of multiple stakeholders, the co-designed service has been adapted to suit patients’ needs and healthcare practices, which may increase its feasibility and acceptance when implemented into practice. In future, the model may inspire pharmacy service planners and researchers to conduct further groundwork and research aimed at developing the CPS and the implementation program. Furthermore, the model may serve to prompt individual pharmacies (i.e., pharmacy owners or managers) or leading organizations (e.g., professional, scientific or governmental organizations) to make decisions for adapting the CPS to their particular context and pilot the service in a real-world setting.

## Additional files

Additional file 1: Focus group and interview guide used to direct the discussions with patients. It encompasses the topics that were addressed as part of the focus group and interviews with patients (in step 1) along with the general questions and prompts used by the facilitator. (DOCX 20 kb)

Additional file 2: Focus group guide used to direct the discussions with healthcare professionals and pharmacists. It encompasses the topics that were addressed as part of the focus group and interviews with the mixed group of healthcare professionals (in step 2) and with pharmacists (in step 3) along with the general questions and prompts used by the facilitator. (DOCX 19 kb)

Additional file 3: Selected quotes from participants. Selection of quotes that support the statements in the result section. (DOCX 21 kb)

## Abbreviations

AF: Atrial fibrillation
BP: Blood pressure
CPS: Community pharmacy service
GP: General Practitioner

## References

[1] Kirchhof P, Breithardt G, Bax J (2016). A roadmap to improve the quality of atrial fibrillation management: proceedings from the fifth atrial fibrillation network/European heart rhythm association consensus conference. Europace.

[2] Ball J, Carrington MJ, McMurray JJ, Stewart S (2013). Atrial fibrillation: profile and burden of an evolving epidemic in the 21st century. Int J Cardiol.

[3] Deloitte Access Economics Pty Ltd. Off beat: Atrial fibrillation and the cost of preventable strokes. 2011. https://www.baker.edu.au/Assets/Files/Off%20Beat%20-%20Atrial%20fibrillation%20and%20the%20cost%20of%20preventable%20strokes_Sep%202011.pdf. Accessed 02 Feb 2017.

[4] Gattellari M, Goumas C, Aitken R, Worthington JM (2011). Outcomes for patients with ischaemic stroke and atrial fibrillation: the PRISM study (a program of research informing stroke management). Cerebrovasc Dis.

[5] Freedman B, Potpara TS, Lip GY (2016). Stroke prevention in atrial fibrillation. Lancet.

[6] Ferguson C, Inglis SC, Newton PJ, Middleton S, Macdonald PS, Davidson PM (2016). Education and practice gaps on atrial fibrillation and anticoagulation: a survey of cardiovascular nurses. BMC Med Educ.

[7] Stergiou GS, Karpettas N, Protogerou A, Nasothimiou EG, Kyriakidis M (2009). Diagnostic accuracy of a home blood pressure monitor to detect atrial fibrillation. J Hum Hypertens.

[8] Wiesel J, Fitzig L, Herschman Y, Messineo FC (2009). Detection of atrial fibrillation using a modified microlife blood pressure monitor. Am J Hypertens.

[9] Verberk WJ, Omboni S, Kollias A, Stergiou GS (2016). Screening for atrial fibrillation with automated blood pressure measurement: research evidence and practice recommendations. Int J Cardiol.

[10] Parati G, Stergiou GS, Asmar R (2010). European Society of Hypertension practice guidelines for home blood pressure monitoring. J Hum Hypertens.

[11] Pickering TG, White WB, American Society of Hypertension Writing Group (2008). ASH position paper: home and ambulatory blood pressure monitoring. When and how to use self (home) and ambulatory blood pressure monitoring. J Clin Hypertens (Greenwich).

[12] Ayorinde AA, Porteous T, Sharma P (2013). Screening for major diseases in community pharmacies: a systematic review. Int J Pharm Pract.

[13] Fikri-Benbrahim N, Faus MJ, Martinez-Martinez F, Alsina DG, Sabater-Hernández D (2012). Effect of a pharmacist intervention in Spanish community pharmacies on blood pressure control in hypertensive patients. Am J Health Syst Pharm.

[14] Sendra-Lillo J, Martinez-Martinez F, Garcia-Corpas JP, Marin Rivas F, Sabater-Hernández D (2015). Validity of home blood pressure measurements manually registered by patients after an educational session provided by community pharmacists. Blood Press Monit.

[15] Sabater-Hernández D, Sabater-Galindo M, Fernandez-Llimos F (2016). A systematic review of evidence-based community pharmacy services aimed at the prevention of cardiovascular disease. J Manag Care Spec Pharm.

[16] Lowres N, Neubeck L, Salkeld G (2014). Feasibility and cost-effectiveness of stroke prevention through community screening for atrial fibrillation using iPhone ECG in pharmacies. The SEARCH-AF study. Thromb Haemost.

[17] Twigg MJ, Thornley T, Scobie N (2016). Identification of patients with atrial fibrillation in UK community pharmacy: an evaluation of a new service. Int J Clin Pharm.

[18] Mossialos E, Courtin E, Naci H (2015). From "retailers" to health care providers: transforming the role of community pharmacists in chronic disease management. Health Policy.

[19] Franco-Trigo L, Hossain LN, Durks D (2017). Stakeholder analysis for the development of a community pharmacy service aimed at preventing cardiovascular disease. Res Social Adm Pharm.

[20] Sabater-Hernández D, Moullin JC, Hossain LN (2016). Intervention mapping for developing pharmacy-based services and health programs: a theoretical approach. Am J Health Syst Pharm.

[21] Perrott BE (2013). Including customers in health service design. Health Mark Q.

[22] Donetto S, Pierri P, Tsianakas V, Robert G (2015). Experience-based co-design and healthcare improvement: realizing participatory Design in the Public Sector. Des J.

[23] Elf M, Frost P, Lindahl G, Wijk H (2015). Shared decision making in designing new healthcare environments-time to begin improving quality. BMC Health Serv Res.

[24] Villanueva R, Gugel D, Dwyer DM (2013). Collaborating across multiple health care institutions in an urban colorectal cancer screening program. Cancer.

[25] Bartholomew LK, Markham CM, Ruiter RAC, Fernández ME, Kok G, Parcel GS. Planning health promotion programs: an intervention mapping approach. 4th ed. San Francisco, CA: Jossey-Bass; 2016.

[26] Waugh A, Austin A, Manthorpe J (2013). Designing a complex intervention for dementia case management in primary care. BMC Fam Pract.

[27] Boyd H, McKernon S, Mullin B, Old A (2012). Improving healthcare through the use of co-design. N Z Med J.

[28] Wherton J, Sugarhood P, Procter R, Hinder S, Greenhalgh T (2015). Co-production in practice: how people with assisted living needs can help design and evolve technologies and services. Implement Sci.

[29] Kelly L, Caldwell K, Henshaw L (2006). Involving users in service planning: a focus group approach. Eur J Oncol Nurs.

[30] Bazeley P (2009). Analysing qualitative data: more than ‘identifying themes’. Malays J Qual Res.

[31] Flottorp SA, Oxman AD, Krause J (2013). A checklist for identifying determinants of practice: a systematic review and synthesis of frameworks and taxonomies of factors that prevent or enable improvements in healthcare professional practice. Implement Sci.

[32] Jones MI, Greenfield SM, Bray EP (2012). Patients' experiences of self-monitoring blood pressure and self-titration of medication: the TASMINH2 trial qualitative study. Br J Gen Pract.

[33] Kannel WB, Wolf PA, Benjamin EJ, Levy D (1998). Prevalence, incidence, prognosis, and predisposing conditions for atrial fibrillation: population-based estimates. Am J Cardiol.

[34] Lowres N, Krass I, Neubeck L (2015). Atrial fibrillation screening in pharmacies using an iPhone ECG: a qualitative review of implementation. Int J Clin Pharm.

[35] Lowres N, Neubeck L, Freedman SB (2016). Can screening for atrial fibrillation be implemented at scale?. Europace.

[36] McCabe PJ (2011). What patients want and need to know about atrial fibrillation. J Multidiscip Healthc.

[37] Grant S, Hodgkinson JA, Milner SL (2016). Patients' and clinicians' views on the optimum schedules for self-monitoring of blood pressure: a qualitative focus group and interview study. Br J Gen Pract.

[38] Tsakiri C, Stergiou GS, Boivin JM (2013). Implementation of home blood pressure monitoring in clinical practice. Clin Exp Hypertens.

[39] Green LW, Kreuter MW (2005). Health program planning: an educational and ecological approach.

[40] Michie S, van Stralen MM, West R (2011). The behaviour change wheel: a new method for characterising and designing behaviour change interventions. Implement Sci.

[41] Sharman JE, Howes FS, Head GA (2015). Home blood pressure monitoring: Australian expert consensus statement. J Hypertens.

[42] Krause J, Van Lieshout J, Klomp R (2014). Identifying determinants of care for tailoring implementation in chronic diseases: an evaluation of different methods. Implement Sci.

[43] Berbatis C, Sunderland V, Joyce A, Bulsara M, Mills C (2007). Enhanced pharmacy services, barriers and facilitators in Australia's community pharmacies: Australia's National Pharmacy Database Project. Int J Clin Pharm.

[44] Roberts A, Benrimoj S, Chen T, Williams K, Aslani P (2006). Implementing cognitive services in community pharmacy: a review of facilitators used in practice change. Int J Clin Pharm.

[45] Powell BJ, Waltz TJ, Chinman MJ (2015). A refined compilation of implementation strategies: results from the expert recommendations for implementing change (ERIC) project. Implement Sci.

[46] Hoffmann TC, Glasziou PP, Boutron I (2014). Better reporting of interventions: template for intervention description and replication (TIDieR) checklist and guide. BMJ.

