# Supplementary material for: A stakeholder co-design approach for developing a community pharmacy service to enhance screening and management of atrial fibrillation
Source: BMC Health Serv Res. 2018 Feb 27;18:145. doi: 10.1186/s12913-018-2947-7 (PMC6389098; doi:10.1186/s12913-018-2947-7)
Supplement: Supplementary file 1 — Focus group and interview guide used to direct the discussions with patients. It encompasses the topics that were addressed as part of the focus group and interviews with patients (in step 1) along with the general questions and prompts used by the facilitator. (DOCX 20 kb) [file 12913_2018_2947_MOESM1_ESM.docx]

Additional file 1. Focus group and interview guide used to direct the discussions with patients

| **Themes (topics to be explored)** | **Broad descriptive questions** | **Possible questions, probes, prompts** |
| --- | --- | --- |
| 1. Awareness/knowledge of atrial fibrillation (AF) | What do you know about AF?  (For those with AF/with prior knowledge): How did you find out about AF? | Causes, consequences  Disease trajectory  Risk factors and management re disease progression  Symptoms, recurrent episodes  Treatments options; rate-control vs rhythm-control  Action plans  Management strategies  Monitoring  When to seek help  Impact on life/other conditions/stress, psychosocial |
| 2. Experiences regarding (AF), self-management and self-monitoring | (For those with AF): When did you first know something was wrong?  What is it like having AF? How/where did you learn what you know?  [Same questions for those without AF but with hypertension] | Symptoms  Test results  Diagnosis  Expense - Medical appointments, monitoring, medication  Accommodating illness, e.g.: manage fatigue, dyspnoea  Stress/anxiety  Inconvenience  What it means for their day-to-day life |
|  | What do you do to look after this condition? What do your health professionals do to help you to manage AF? | Medication, ablation  Rest, sleep management  Stress management, treat depression, etc.  Monitoring  Assistance from carers/family members  GP/pharmacist/specialist/RDNS/home carer, etc |
|  | There are devices that allow people to detect and monitor AF/hypertension. Has anyone used one of these?  (Those who haven't used): what do you know about them?  (Those who have used): How are they to use? | Which specific devices  Purposes/advantages of monitoring  How to use  Portability  Cost  Readability  Interpretation |
| 3. Tailoring health services to the individuals | If a service to monitor AF was designed specifically for you, what would it need to include?  What would a ‘good’ experience with AF diagnosis look like?  What would a ‘good’ experience with AF monitoring look like?  What do you need to know about AF?  How can healthcare professionals help you to know more about the problem? | Pharmacy-based, provision for home visiting  Information  Specific health professional expertise  Medical treatment  Access to and availability of service  Specific supplies, equipment, materials |
| Research team introducing the self-monitoring device and related educational materials. Open discussion with the participants. | | |
| 4. Participant Needs | What are your first thoughts?  What would encourage you to use it?  If you were going to use this device, what would you need?  What would be the best way for you to learn about [how to use it, the benefits, etc – as raised by participants]? | How to use it  How often to use  Durability  Maintenance  Portability  Reading/interpretating information  Recording/retrieving information  Sharing information with HPs |
| 5. Acceptability of an AF service in community pharmacy | (Community pharmacists also provide health services to the population, such as Home Medication Review, MedsCheck, Diabetes MedsCheck, etc.)  What kind of role do community pharmacists have regarding your health?  How could they help you with a health problem such as atrial fibrillation? | Advantages  Disadvantages  Potential role  Experiences of community pharmacists  Local services vs specialist service  Type of pharmacy: Chemist Warehouse, independent, Tony White  Pre-existing relationship, i.e.: regular pharmacy/pharmacist |
| 6. Cost | Would you be willing to pay for the device?  What would be an acceptable amount to pay for such a device?  Would you be willing to pay for the service provided by a pharmacist? | Cost of the device: $150 – 170  Service would include: educational session; taking BP; show you how to use and maintain the device |
| 7. Integration of pharmacy services into the healthcare system | If healthcare services were ‘working together’, what does that mean to you?  Have you ever experienced your pharmacist and other healthcare professionals 'working together'?  If pharmacists and other healthcare professionals were to ‘work well’ together for you, what would need to happen? | Privacy  Information sharing between pharmacy staff, GPs, others  Communication between HPs  Service accessibility  Service remuneration  Patients want to know the outcome/results  “Busyness” of pharmacies; feeling ‘rushed’  Will they offend GPs by participating?  Do they have to go back to GP? |
| Final open question: Any other issues about atrial fibrillation or hypertension you would like to raise? | | |
