# Supplementary material for: A stakeholder co-design approach for developing a community pharmacy service to enhance screening and management of atrial fibrillation
Source: BMC Health Serv Res. 2018 Feb 27;18:145. doi: 10.1186/s12913-018-2947-7 (PMC6389098; doi:10.1186/s12913-018-2947-7)
Supplement: Supplementary file 2 — Focus group guide used to direct the discussions with healthcare professionals and pharmacists. It encompasses the topics that were addressed as part of the focus group and interviews with the mixed group of healthcare professionals (in step 2) and with pharmacists (in step 3) along with the general questions and prompts used by the facilitator. (DOCX 19 kb) [file 12913_2018_2947_MOESM2_ESM.docx]

Additional file 2. Focus group and interview guide used to direct the discussions with healthcare professionals and pharmacists

| **Themes (topics to be explored)** | **Broad descriptive questions** | **Possible questions, probes, prompts** |
| --- | --- | --- |
| 1. Target population of the AF community pharmacy service | Thinking about the feasibility and usefulness of the service…  Of your patient population, whom do you think would most benefit from this service? | Depending on their profile, potential service users can have different:   - Awareness and knowledge regarding AF - Concern about AF - Willingness to self-screening/self-monitoring AF, to pay for the device, to receive the service   Potential targets groups of the service are:   - Individuals with AF - Individuals without AF with other cardiovascular conditions (e.g., hypertension); without any other cardiovascular conditions (‘healthy individuals) |
| Research team introducing the self-monitoring device and related educational materials. Open discussion with the participants. | | |
| 2. Service components | How does a high-quality pharmacy service for the targeted population look like? Please, provide some detail about any proposed strategy, method, technique, process, protocol, support material, educational content, etc. | Assessments conducted by the pharmacist (e.g., clinical measurements, risk assessment, adherence to guidelines, etc.…)  Actions targeting patients (e.g., education)  Actions targeting other healthcare professionals (e.g., recommendations)  Patient referral  Patient follow-up |
| 3. Service integration into the healthcare system | Considering your current service provision and current healthcare practices…  What should be taken into account to integrate this service into practice? Please, provide some detail about any proposed strategy, method, technique, process, protocol, support material, educational content, etc. | Service remuneration & other professional incentives  Marketing and publicity, recruitment processes  Pharmacy staff capacity & training, resources  Workflow  Professional organizations support, policy environment  Marketing and publicity, recruitment processes  Privacy  Relationships with other health professionals and services |
