# Supplementary material for: A stakeholder co-design approach for developing a community pharmacy service to enhance screening and management of atrial fibrillation
Source: BMC Health Serv Res. 2018 Feb 27;18:145. doi: 10.1186/s12913-018-2947-7 (PMC6389098; doi:10.1186/s12913-018-2947-7)
Supplement: Supplementary file 3 — Selected quotes from participants. Selection of quotes that support the statements in the result section. (DOCX 21 kb) [file 12913_2018_2947_MOESM3_ESM.docx]

| Additional file 3. Selected quotes from participants | |
| --- | --- |
| Q1 | *“I’m thinking of the person who knows nothing about this. They’ve had some pressure in the chest… maybe they go to the doctor and it’s up on the wall there. They’ll wander over and think: ‘What’s an arrhythmia?’ They’re not all the same…and there’s nothing to say AF, Atrial Fibrillation, is an abnormal heartbeat. And that’s the first thing. Because anybody that doesn’t know about the medicine but they’ll see ‘An irregular heartbeat’.*  Participant with hypertension and potential AF (interview with potential service users) |
|  | *“Well, as somebody who was a specialist reading teacher in high school for 30-odd years, the reading level on these requires a university level education. So, for putting around the place for the information of the general public they’re really not very useful. If I couldn’t read it I would ignore it”.*  Participant without AF and without hypertension (focus group with potential service users) |
| Q2 | *“When I had those AF episodes they were frightening and I didn’t think about deep breathing or anything, especially the time I lost my hearing and next the eyesight, I just thought of getting home as fast as I could. But in this last lot of time I’ve had a lot of tensions in the last year and I had to do a lot of deep breathing. And I was in AF and I didn’t realise it, and my husband was so ill. And I was in AF then. And then I went to the cardiologist and then I found out how bad things were. But I was then - and I still am now - trying to control my emotion as it comes up with this deep breathing. And it does relax me… But at times lately, I haven’t been sure whether I’m having these physical symptoms because my husband died or because I’m in AF”.*  Participant with AF, without hypertension (focus group with potential service users) |
| Q3 | *“When I had those [AF] episodes, they were frightening and I didn’t think about deep breathing or anything… But in this last lot of time I’ve had a lot of tensions in the last year and I had to do a lot of deep breathing. And I was in AF and I didn’t realise it, and my husband was so ill. And I was in AF then. By then I knew what to look out for, but then I went to the cardiologist and then ‘woof’ I found out how bad things were. But I was then - and I still am now - trying to control my emotion as [AF] comes up with this deep breathing.”*  Participant with AF, without hypertension (focus group with potential service users) |
| Q4 | *“This was quite frightening - I was in the church actually and suddenly I lost my hearing. I just couldn’t hear a thing. […]And then the next thing my eyesight went. I was very frightened and I thought: No, if I stand I might drop. I didn’t know what to do, so I just sat there through the whole thing… In this situation now I’d call the cardiologist. Or I’d ring an ambulance to go straight to the casualty of RPA Hospital because they’ll take you from there. They will notify him because he’s at RPA and he would be down there”.*  Participant with AF, without hypertension (focus group with potential service users) |
| Q5 | *“I don’t know whether something could help to the AF; I don’t know. But by nature and my own experience and I noticed that deep breathing… or just getting what is out of the way, I think it could have been helping. […] This monitor would help work out what is stress, what is AF, if what I do to help stress helps AF”.*  Participant with hypertension and AF (focus group with potential service users) |
| Q6 | *“Well I feel that I am very well looked after. And I’ve never had any real problems with it, so it’s really not high up the list. The arthritis stops me from doing a hell of a lot more than the AF does”.*  Participant with AF, without hypertension (interview with potential service users) |
| Q7 | *Oh yes, I suppose the novelty… I could test [my wife’s] and I could test mine. Anyone that came to the house I could test their blood pressure. […] Do I really need to do this monitoring business? Maybe years ago when I’d just found out I had high blood pressure and we needed to control it and I was worried. But it’s been under control for so long now without having to fork out the big dollars you’re asking for it, so I’m not about to start now.*  Participant with hypertension, without AF (interview with potential service users) |
| Q8 | *“Well, it’s sensible but you could fill your entire house with medical testing equipment, couldn’t you? I mean you could spend your life testing on and off, for everything you don't have”.*  Participant without AF and without hypertension (focus group with potential service users) |
| Q9 | *“No, I’d be happy about that, I think it would be great to find out straight away. I wouldn’t be concerned about a pharmacist telling me I needed to see a doctor because of something he sees on the readings. I’ve been around the block, I know you often have to see this, that and the other and have a multitude of tests and appointments sometimes, before you get told ‘you have this”.*  Participant with hypertension, without AF (focus group with potential service users) |
| Q10 | *“I’d be very dubious about having one of these expensive devices prescribed for me by a chemist, by a pharmacist. I would want a doctor to say: ‘You need this. Here is a script. You know, take this to your pharmacist’. And then you get the device”.*  Participant with hypertension, without AF (interview with potential service users) |
| Q11 | *“They have to keep privacy no matter if it’s something serious or small… It’s something ethical and moral. That’s what they should learn also in university”.*  Participant with hypertension, without AF (focus group with potential service users) |
|  | *“And also the chemist would have to be very forthright in the beginning and say: ‘Before we start, what we’re going to cover here it’s just between you and me now’. And it should be explained or giving them a pamphlet to read that anything that is disclosed that the patient should also sign that he or she agrees that this is to be passed onto the doctor”.*  Participant with AF, without hypertension (focus group with potential service users) |
| Q12 | *“And that’s one of the problems you’re going to face with the chemist because the idea of taking your blood pressure for free is what brings them into the shop, that’s what they’re there for. See, like they do Diabetes’ sugar-level test and everything these days there. Yeah, they’re sort of turning into a lay doctor where you can go and have it done for free”*  Participant with potential AF and hypertension (interview with potential service users) |
| Q13 | *“People who have been in AF, because you can record it. If you were prescribing for that person to manage their hypertension, it would be nice to be able to see where it was going if they’re the high risk ones with hypertension. Also, those one with the AF, they’re very likely to go back into AF again, so you’d want to be able to watch those people. Or any AF people who have been AF in the past but not in AF anymore”.*  Pharmacy owner (focus group with mixed stakeholders) |
|  | *“Purchasing the machine, I’ve just got a little problem with it because I just don’t know whether it’s going to be of any particular value unless we’re targeting high-risk people that have got hypertension and are more elderly”.*  General Practitioner (focus group with mixed stakeholders) |
| Q14 | **Cardiologist:** *And that kind of language really isn’t helpful… I don’t think I’ve ever diagnosed a patient with AF who knew what ‘atrial fibrillation’ is.*  **Pharmacist:** *I think most of my patients would walk past a poster [that used that term]. I wouldn’t put it up.*  (Focus group with mixed stakeholders) |
| Q15 | *“A lot of the information that we would be thinking of providing to people would be about modifiable lifestyle factors, and it really depends on that person. Some people might like hardcopies. They actually like booklets, pamphlets – that sort of thing. Other people just say: ‘Just direct me to a podcast. I’ll listen to it. I want to look on the website’. So, it really depends on that person. I think if we had … a collection of endorsed collateral that we could provide, then I think that would be really useful”.*  Nurse practitioner (focus group with mixed stakeholders) |
| Q16 | *“[Home monitoring results] need to be validated by an ECG because it’s just a pulse essentially. But the more you look, the more you will find, or the longer you look, the longer you will find. So, you could go for two weeks or you could go for a month. I would suggest going for a month if you were doing it, because the only study that’s looked at this intermittent one is the [Swedish study] that in 7,000 people used it for two weeks. So, it would be quite interesting to go for a month and get extra information”.*  Cardiologist (focus group with mixed stakeholders) |
| Q17 | *“What is unique about this device is, it’s giving the knowledge that people need to seek appropriate care. But part of the service needs to be to say something that they need to have an ECG, which most people have heard of, but stipulate that this is not a diagnosis. It has to be verified”.*  Cardiologist (focus group with mixed stakeholders) |
| Q18 | *“We’ve been using the (BP & AF) machines in our pharmacy for about eight months’ now. In terms of provision of the service, at the beginning I thought okay, what we will do is we will have the free blood pressure monitoring service and then if anyone wants to use the machine we’ll charge $5. That hasn’t happened. And as it turned out, within the first three or four months we were just introducing patients to this particular blood pressure machine… But we have now sold five in the last six months. So people who have come back to our store at a regular basis to use it - there might be about 20, 30 people who have been doing that - five of them have bought it”.*  Pharmacist (focus group with community pharmacy owners and managers) |
| Q19 | *“So, could it not run as almost like a screen in the pharmacy, rent out the device for a week, get them on board, do the downloads, start that process, then you have a bit of a buy in in terms of them choosing to purchase the machine? I think with the company sponsoring it, obviously ultimately they want the sale of the machine, but not everyone is going to need $170 machine straight out. So, maybe the offering needs to be hire out, because I don’t think running it in the pharmacy is going to give you best practice to pick it up, but offering it to them to take it for a week, take it for two weeks, maybe that will increase detection rates. They might not have any attacks in that period, but maybe it’ll improve the people that we are picking up a little bit more”.*  Pharmacy owner (focus group with mixed stakeholders) |
| Q20 | *“Why would they come back to us? Then the GP has got to get the blood pressure reading and they go to them and they don’t come back. It’s just a transaction, it just ends up being a simple transaction and that’s it… We do that, that’s our one-off payment, they take the machine, then we’re completely cut out. They get the referral, they go to the doctor; they don’t come back to us regarding this at all. This is a one-off”.*  Pharmacist (focus group with community pharmacy owners and managers) |
| Q21 | *“Say you’ve monitored a person with AF and you’re the pharmacist, you’ve taken them to their room, you’ve given the pamphlet, you say: ‘Go and see your GP. It’s probably pretty important’. And you ring them up a week later: ‘How’d you go with the GP?’; ‘I haven’t been yet’. So, you need parameters around that as well”.*  Nurse practitioner (focus group with mixed stakeholders) |
| Q22 | *“I feel that it’s not good enough that to detect a patient with AF with the machine and you just send them off on their merry way without any feedback. You say: ‘Oh, go and see the GP’. But then there’s no feedback loop to say they’ve actually gone there, you know? It would be nice to have, like, a flow-chart to make sure that if you refer them to a GP, there’s some follow-up or there’s some connection - the next step. Rather than, as soon as they leave the clinical room, you have no idea where, whether they go away and follow-up or not”.*  Pharmacist (focus group with community pharmacy owners and managers) |
| Q23 | *“I think it’s got to be done by pharmacists. You can’t have it both ways…. you can’t delegate it on, because otherwise you’re going to lose that professionalism. That’s very important. I’m speaking as a pharmacist and a GP – I’ve done both – so it’s very important to maintain that role. You can’t delegate it to anyone else. You’ve got to do it yourself. If you can’t do it, you don’t do it, or you employ someone else to do it. That’s my strong opinion”.*  General Practitioner (focus group with mixed stakeholders) |
| Q24 | ***Cardiologist:*** *“Do you think you should limit it to pharmacists with a room or a space?”*  ***Pharmacy manager:*** “*I think so. It shouldn’t be a moneymaking thing for a pharmacy to get as many people as you can. I think to do it properly, yeah. If it takes 15 minutes to do the whole thing and they should be sitting”.*  (Focus group with mixed stakeholders) |
| Q25 | *“It is good to have services in the pharmacy, but then you do need two pharmacists in a pharmacy sometimes, to run the services. And I’m wondering whether small pharmacies eventually will be left on the wayside because they can’t afford to have two pharmacists on. And if you don’t have a clinical room, you don’t have to keep up to standards with a lot of things because you’re not enrolled in all these services. So I’m wondering whether that could be an issue in pharmacy down the line”.*  Pharmacist (focus group with community pharmacy owners and managers) |
| Q26 | *“… as for small pharmacies not being able to compete: rubbish. Small pharmacies as a general rule will be much more able to compete if they design their systems and procedures to cope… … you’re a lot more agile if you’re smaller. You want to introduce … AF testing, 20 minutes, you’re on your way! It’s a different ball-game and we need to encourage small pharmacies”*.  Pharmacist (focus group with community pharmacy owners and managers) |
| Q27 | *“Pharmacists, ideally, should speak to the practice doctors and say: ‘I’m about to start to do this. How can I best communicate to you?’ On a personal level, a lot of pharmacists do ring doctors.*  Cardiologist (focus group with mixed stakeholders) |
| Q28 | *“I think the optimal is for the pharmacy that is going to take this one, they would have a range of maybe five to 10 practices that they regularly get people coming in for their scripts. Pharmacists, ideally, should speak to the practice doctors and say: ‘I’m about to start to do this. How can I best communicate to you?’ … The second thing is you should say: ‘We’re going to throw up some of these things which won’t be atrial fibrillation, and then we’ll throw up some things that are. What’s our best of communication with you?’ One they get some sort of warning, or a fax, or an e mail, or something, they’ll be duty-bound to do something about it”.*  General Practitioner (focus group with mixed stakeholders) |
| Q29 | *“We do coeliac screening testing in our pharmacy and initially we had a lot of negative feedback in the media and everything else. But a few of the doctors that I know in person, when I’ve explained to them that: ‘Look, you’re too busy. This is screening people to direct them towards you. The only other option for these people is to self-mediate, go to health food shops or to try and Dr. Google it, but as a team we have the opportunity. They haven’t been able to communicate it to the GP’s, they’ve come and talk to the pharmacist, allowed the pharmacist to screen them and direct them back for treatment’. When you talk to [GPs] like that, they actually see the big picture; they realize that pharmacists can be part of the team to correctly direct the patients towards the GP’s, rather than towards Google or towards health products, complimentary products that may or may not help”.*  Pharmacist (focus group with community pharmacy owners and managers) |
| Q30 | *“We have done hundreds of pharmacist-administered flu vaccines in our two shops this year, and [the software program we are using] has got a section, ‘Would you like us to write to or inform your GP you’ve had the flu vaccine?’ Not a single person has said ‘Yes’. They just go, ‘I don't care if my GP doesn't know, if I see them next time I'll let them know’. But if you asked the GP’s they will say: ‘No, the administration has to make sure that you tell the GP’. Why? It’s a flu vaccine? It’s a barrier that we're trying to break and it's just program after program”.*  Pharmacist (focus group with community pharmacy owners and managers) |
| Q31 | *“You know, it’s very good with my pharmacist now, because I’ve been going to the same place for a long time. He and the girl know all of my problems and they know my history, you know? But that took years, where maybe a chat with my doctor would have told them everything they need to know early on. See, I’m not a doctor or a pharmacist or a nurse or anything, so I don’t know all of what I need to tell them”.*  Participant with AF and hypertension (interview with potential service users) |
| Q32 | *“I know how in pharmacies that you often see they have: ‘The baby health nurse is going to be here on Friday mornings’ or whatever. Is there an option for another clinician to come in in the very busy pharmacies, like a nurse, to come in and do those spot checks for your patients? Even the practice nurse from the GP could come to the pharmacy and screen patients there just for a different option”.*  Stroke Foundation representative (focus group with mixed stakeholders) |
